# Anti-cancer effect of pristimerin by inhibition of HIF-1α involves the SPHK-1 pathway in hypoxic prostate cancer cells

**DOI:** 10.1186/s12885-016-2730-2

**Published:** 2016-08-31

**Authors:** Seon-Ok Lee, Joo-Seok Kim, Myoung-Sun Lee, Hyo-Jeong Lee

**Affiliations:** 1 Department of Cancer Preventive Material Development, Graduate School, Kyung Hee University, Seoul, Republic of Korea; 2 College of Korean Medicine, Kyung Hee University, 1Hoegi-dong, Dongdaemun-gu, Seoul 130-701 Republic of Korea; 3 Department of Science in Korean Medicine, Graduate School, Kyung Hee University, Seoul, Republic of Korea

**Keywords:** Hypoxia, Pristimerin, SPHK-1, Prostate cancer, HIF-1α

## Abstract

**Background:**

Hypoxia is a typical character of locally advanced solid tumours. The transcription factor hypoxia-inducible factor 1α (HIF-1α) is the main regulator under the hypoxic environment. HIF-1α regulates various genes to enhance tumour progression, angiogenesis, and metastasis. Sphingosine kinase 1 (SPHK-1) is a modulator of HIF-1α.

**Methods:**

To investigate the molecular mechanisms of pristimerin in association with SPHK-1 pathways in hypoxic PC-3 cancer cells. Vascular endothelial growth factor (VEGF) production, cell cycles, and SPHK-1 activity were measured, and western blotting, an MTT assay, and an RNA interference assay were performed.

**Results:**

Pristimerin inhibited HIF-1α accumulation in a concentration- and-time-dependent manner in hypoxic PC-3 cells. Pristimerin suppressed the expression of HIF-1α by inhibiting SPHK-1. Moreover, inhibiting SPHK-1 with a sphingosine kinase inhibitor enhanced the suppression of HIF-1α, phosphorylation AKT, and glycogen synthase kinase-3β (GSK-3β) by pristimerin under hypoxia. Furthermore, a reactive oxygen species (ROS) scavenger enhanced the inhibition of HIF-1α and SPHK-1 by pristimerin.

**Conclusion:**

Taken together, these findings suggest that pristimerin can exert an anti-cancer activity by inhibiting HIF-1α through the SPHK-1 pathway.

**Electronic supplementary material:**

The online version of this article (doi:10.1186/s12885-016-2730-2) contains supplementary material, which is available to authorized users.

## Background

Hypoxia is a common characteristic of locally advanced solid tumours [1] and up to 50–60 % of solid tumours include areas of hypoxic tissues [2]. The hypoxic tumour contributes to aggressive and metastatic cancer phenotypes that are associated with resistance to radiation therapy, chemotherapy, and a poor treatment outcome [3, 4].

The hypoxia inducible factor-1 (HIF-1) is a transcription factor and also a key factor that maintains oxygen homeostasis in mammalian cells [5]. HIF-1 is a heterodimer consisting of HIF-1α and β subunits [6]. HIF-1α is dominantly expressed under hypoxic conditions, however, it exists in low levels under normoxic conditions [7]. On the contrary, HIF-1β is expressed constitutively [7]. In normoxic conditions, HIF-1α is hydroxylated by a tumour suppressor Von Hippel-Lindau (VHL) protein of the E3 ubiquitination ligase complex. Whereas, under hypoxic conditions, HIF-1α remains unhydroxylated and facilitates several factors, [8–10] such as angiogenesis, tumour proliferation, tumour survival, and glycolysis [11, 12]. Sphingosine-1-phoshate (S1P) is a signaling sphingolipid metabolite and a potent lipid mediator, which regulates progress in tumour cells such as cell growth, proliferation, apoptosis, invasion, angiogenesis, calcium homeostasis, and vascular maturation [13, 14]. S1P precursors generate from sphingosine by sphingosine kinase 1 (SPHK-1), and the generation of S1P precursors triggers either a cell’s proliferation or death [13]. SPHK-1 can act as a catalyst for the ATP-dependent phosphorylation of sphingosine, which stimulates a wide array of growth factors, such as PDGF, FGF, EGF, HGF, VEGF, etc. [15–21]. SPHK-1 mRNA is overexpressed in various solid tumours, such as a breast, brain, lung, stomach, colon, kidney, and ovary tumours [22]. Several studies have demonstrated that SPHK-1 controls the level of HIF-1α during hypoxia in cancer cells [23].

Pristimerin is a naturally occurring triterpenoid quinone methide [24, 25]. Several studies have demonstrated that pristimerin is involved in a variety of multiple biological activities related to anti-inflammatory, anti-oxidant, anti-cancer, anti-malarial, and anti-microbial action [26–28]. Also, pristimerin has shown potent anti-cancer effects, including anti-proliferation, anti-migration, anti-angiogenesis, and apoptosis-inducing activity in various cancer cell lines, including glioma, leukemia, breast, lung, and prostate cancer cell lines [24, 25, 29, 30] by inhibiting NF-kB [29, 31–36]. Recently, Zuo, et al. reported that pristimerin has an inhibitory action on hypoxia-mediated metastasis [4]. Nevertheless, the potential effects and the mechanism of pristimerin in hypoxia-mediated cancers still remain unknown.

Here, we demonstrate that pristimerin inhibits HIF-1α via the SPHK-1 signaling pathway in a prostate cancer cell lines. The results we have yielded provide the mechanism for inhibitory action of HIF-1α and angiogenesis by pristimerin in hypoxic prostate cancer cell lines.

## Methods

### Test chemical

Pristimerin (purity: ≥98 % as determined by HPLC) was purchased from Sigma Aldrich (St Louis, MO, USA).

### Cell culture and hypoxia treatment

The human castration-resistant prostate cancer cell lines PC-3 and DU145 cells were preserved in RPMI1640 (Welgene, Daegu, Korea), supplemented with 10 % FBS and 1 % antibiotics (Welgene, Daegu, Korea). The human androgen responsive prostate cancer cell line LNCaP was maintained in RPMI1640, supplemented with 25 % HEPES (Welgene, Daegu, Korea), 10 % FBS and 1 % antibiotics (Welgene, Daegu, Korea). Normoxically conditioned cells were cultured in a 5 % CO_2_ incubator at 37 °C. The cells cultured under hypoxia were grown in a hypoxic chamber (Forma Scientific, Marietta, OH, USA) containing 1 % oxygen, 5 % carbon dioxide, and 94 % nitrogen at 37 °C.

### Cell viability assay

A colorimetric 3-(4,5-Dimethylthiazol-2-yl)-2,5-diphenyltetrazolium bromide (MTT) assay (Sigma, USA) was used to assess cell viability. Cells (1 × 10^4^ per well) were seeded in 96-well plates (SPL Life Science, Korea) and treated with various concentrations (0, 0.047, 0.094, 0.188, 0.375, 0.75, 1.5, and 3 μM) of pristimerin. After 24 h, 50 μL of MTT reagent (1 mg/mL) was added. After incubation for 1 h, optical density was measured by an ELISA-Reader (Tecan, Switzerland) at a wavelength of 570 nm.

### Western blot analysis

The cells were lysed in RIPA buffer (Cell signaling, USA). The protein extract were separated on SDS–polyacrylamide gels and were electrotransferred to a nitrocellulose membrane (GE healthcare life sciences, UK). The membranes were blocked in 5 % non-fat dry milk and probed with primary antibodies for SPHK-1 (Cell signaling, USA), HIF-1α (Novus Biologicals, USA), AKT (Santa Cruz Biotechnology, Santa Cruz, CA, USA), p-AKT (Santa Cruz Biotechnology, Santa Cruz, CA, USA), GSK-3β (Invitrogen, USA), p-GSK-3β (Cell signaling, USA), VEGF (Santa Cruz Biotechnology, Santa Cruz, CA, USA), PCNA (DAKO, USA), PI3K (Millipore, Germany), and β-actin (Sigma-Aldrich, St, Louis, MO, USA) overnight at 4 °C and HRP-conjugated secondary antibodies. Detection of specific proteins was carried out with an enhanced chemiluminescence (ECL) assay (GE Healthcare Life Sciences, UK).

### Sphingosine kinase assay

To measure sphingosine kinase activity, sphingosine kinase activity assay kit (Echelon, Salt Lake City, UT, USA) was used. The Sphingosine kinase activity assay method was previously described in our other study [37, 38]. Protein extracts (30 μg) were reacted in reaction buffers, 100 μM of sphingosine, and 10 μM of ATP, for 1 h at 37 °C, and then to stop the kinase reaction, a luminescence attached ATP detector was added. Lumistar Optima luminometer (BMG LABTECH, Offenburg, Germany) was used to measure kinase activity. All samples were prepared in triplicates and the assay was repeated at least three times.

### Measurement of VEGF production

VEGF ELISA kit (Invitrogen, Carlsbad, CA, USA) was used to assess VEGF levels in pristimerin and/or SKI exposed PC-3 cells. The VEGF production level measurement methods was previously described in our other study [39]. The culture supernatants was added in a 96-well plate, and reacted with dilution buffer and incubation buffer at room temperature for 2 h. The wells were then washed four times with washing buffer, and then biotin conjugate was added to each well at room temperature for 1 h. After washing, the stabilized chromogen was added into each well and reacted for 30 min at room temperature. The density was measured at 450 nm using a microplate reader (Molecular Devices Co., Sunnyvale, CA, USA) after adding 100 μl of the stop solution.

### Cell cycle assay

The cell cycle was determined according to the protocol described previously [40]. Cells were fixed with 75 % ethanol and resuspended in PBS with RNase (1 mg/mL) at 37 °C for 1 h and stained with propidium iodide (PI). The stained cells were analyzed for DNA content by FACS Calibur containing Cell-Quest Software (Becton-Dickinson, Heidelberg, Germany).

### RNA interference experiments

The siRNA transfection method was previously described in our other study [37, 38]. A polyplus siRNA transfection reagent (Illkirch, France) was used to transfect siRNA for the control or SPHK-1 into PC-3 cells. In brief, siRNA (80 pmol) was mixed with a transfection reagent in serum-free media and reacted for 10 min at room temperature. The siRNA/transfection reagent mixture was added to the cells and incubated for 48 h. The medium was changed before the treatment with pristimerin and/or SKI under hypoxia.

### Statistical analysis

The data showed as means ± S.D. (standard deviation) of three replications each experiment in this study. Analysis of variance (ANOVA) was used to assess the significance of differences between groups. *P* <0.05 was considered to indicate statistical significance.

## Results

### Pristimerin decreases cell viability under hypoxia

**Fig. 1:**
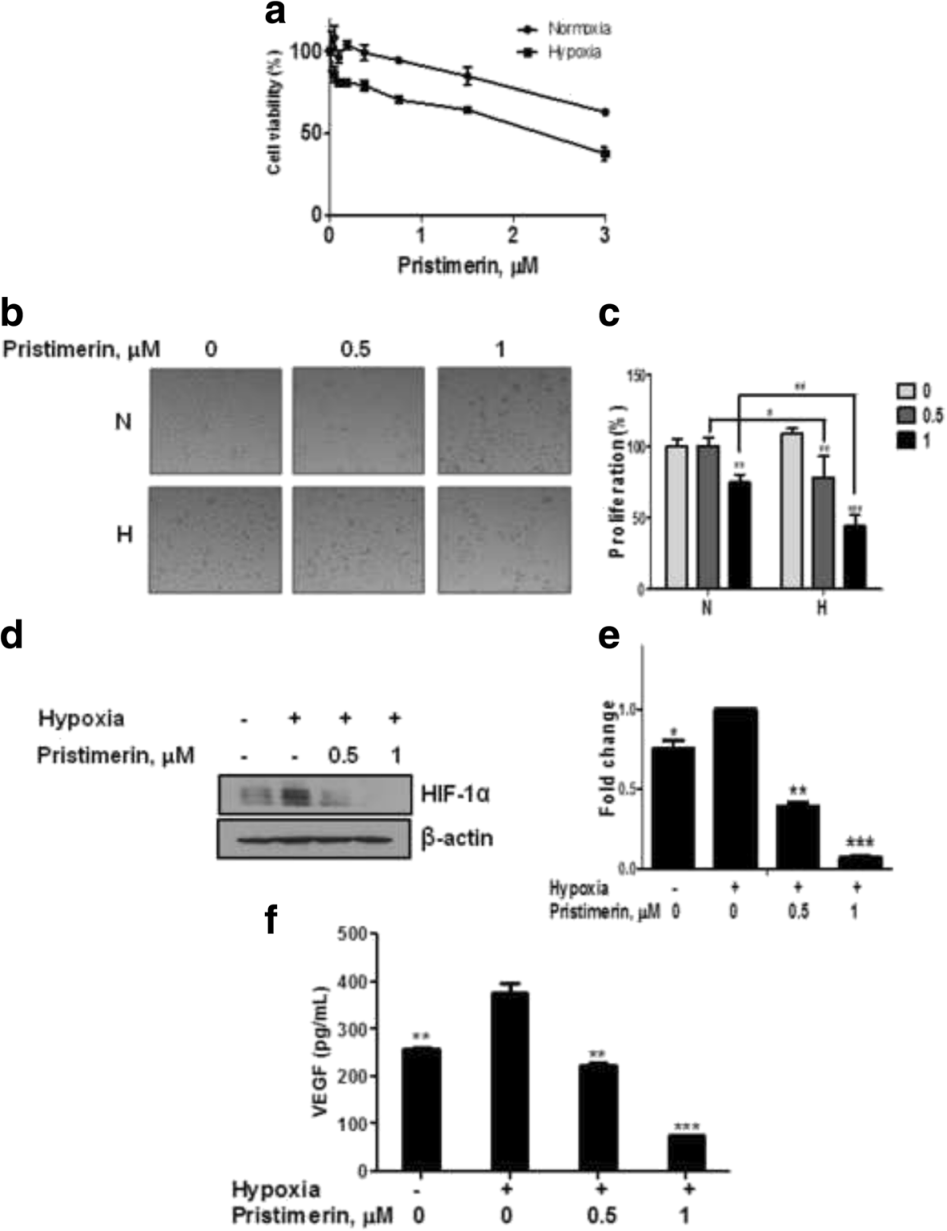
Pristimerin decreases cell viability under hypoxia and inhibits hypoxia-induced HIF-1α. **a** Effects of pristimerin on the cytotoxicity of PC-3 cells for 24 h under normoxic and hypoxic condition. **b** Changes in the morphology of a cell according to the concentration. Cells were treated pristimerin (0, 0.5, and 1 μM) under normoxia and hypoxia for 48 h. **c** Quantitative cell proliferations were shown. The results are expressed as means ± SD for the triplicate. ** p <0.01, *** *p* <0.001 compared with untreated control. *# p* <0.05*, ## p* <0.01 compared with normoxic prestimerin-treated group. **d** Effect of pristimerin on the HIF-1α expression by western blotting. Cells were treated with or without pristimerin (0.5 and 1 μM) under normoxia and hypoxia for 4 h. **e** Quantitative HIF-1α protein levels are shown. The results are expressed as means ± SD for the triplicate. * *p* <0.05, ** *p* <0.01 and *** *p* <0.001 compared with hypoxia control. **f** Effect of pristimerin on the VEGF production. The results are expressed as means ± SD for the duplicate. ** *p* <0.01, *** *p* <0.001 compared with hypoxia control group

To measure whether pristimerin affects cell viability under hypoxic and normoxic conditions, cells were treated with various concentrations of pristimerin in PC-3 cells under hypoxia or normoxia for 24 h. Pristimerin significantly decreased cell viability under hypoxia than it did under normoxia (Fig. 1a). As shown in Fig. 1b and c, pristimerin treatment for 48 h reduced cell growth in hypoxic PC-3 cells. Similar to the 24 h data, pristimerin significantly decreased cell growth under hypoxia more than normoxia.

### Pristimerin decreases HIF-1α abundance and VEGF secretion

To examine whether pristimerin inhibits hypoxia-induced HIF-1α, pristimerin was treated into PC-3 cells under hypoxia for 4 h. As shown in Fig. 1d and e, pristimerin decreased HIF-1α abundance. To examine whether hypoxia-induced VEGF secretion is decreased by pristimerin, the VEGF secretion level was measured on a hypoxia-induced PC-3 cell medium, with pristimerin treatment for 24 h. As shown in Fig. 1f, the VEGF secretion level under hypoxia was higher than under normoxia control. Pristimerin reduced the hypoxia-induced VEGF secretion.

### Pristimerin exerts significant inhibition of SPHK-1 in hypoxic PC-3 cells

**Fig. 2:**
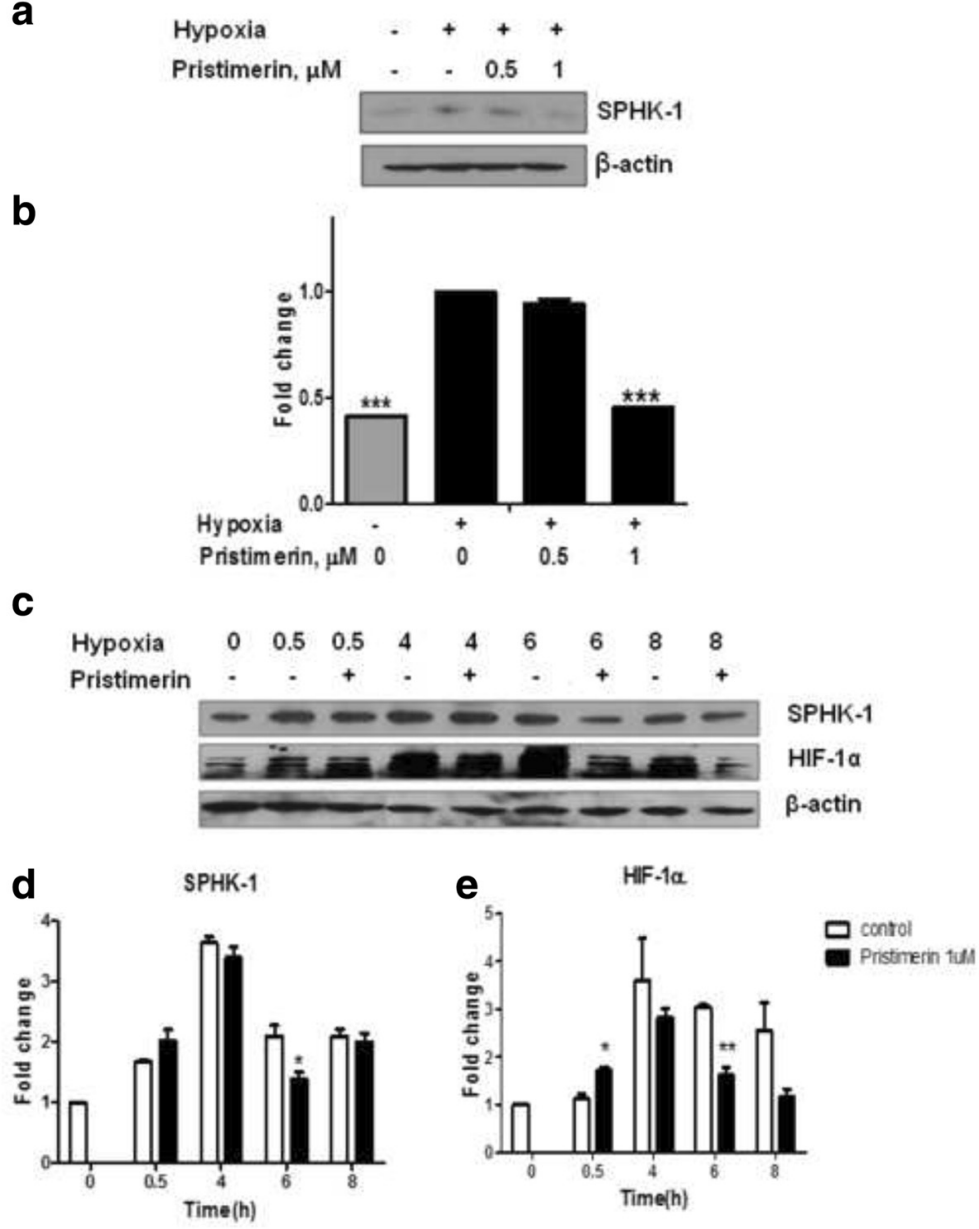
Pristimerin exerts significant inhibition of SPHK-1 in hypoxic PC-3 cells. **a** Cells were treated with or without pristimerin (0.5 and 1 μM) under normoxia and hypoxia for 4 h. Western blotting was performed to determine SPHK-1 expression. **b** Quantitative protein levels are shown. The results are expressed as means ± SD for the triplicate. *** *p* <0.001 compared with hypoxia control. **c** Pristimerin inhibits hypoxia-induced HIF-1α and SPHK-1 accumulation in PC-3 cells under hypoxia in a time-dependent manner. Cells were treated with 1 μM pristimerin for 0, 0.5, 4, 6, or 8 h under hypoxia. Western blotting was performed to determine HIF-1α and SPHK-1 expressions in PC-3 cells. **d** Quantitative protein levels are shown (SPHK-1). The results are expressed as means ± SD for the duplicate. * *p* <0.05 compared with hypoxia control at each time point **e** Quantitative protein levels are shown (HIF-1α). The results are expressed as means ± SD for the duplicate. * *p* <0.05 and ** p <0.01 compared with hypoxia control at each time point

To investigate whether pristimerin affects SPHK-1 in PC-3 cells, the cells were incubated under hypoxia for 4 h with 0.5 or 1 μM of pristimerin. Pristimerin at 1 μM reduced SPHK-1 to 55 % under hypoxia compared with the control (Fig. 2a and b). As SPHK-1 is one of the regulators of HIF-1α, the effect of hypoxia was assessed with the HIF-1α expression. Both the SPHK-1 and HIF-1α accumulation reached the peak 4 h after hypoxia exposure and then decreased in a time-dependent manner. The SPHK-1 and HIF-1α expressions were effectively inhibited by pristimerin (Fig. 2c, d and e).

### SPHK-1 mediates the activation of HIF-1α under hypoxia

**Fig. 3:**
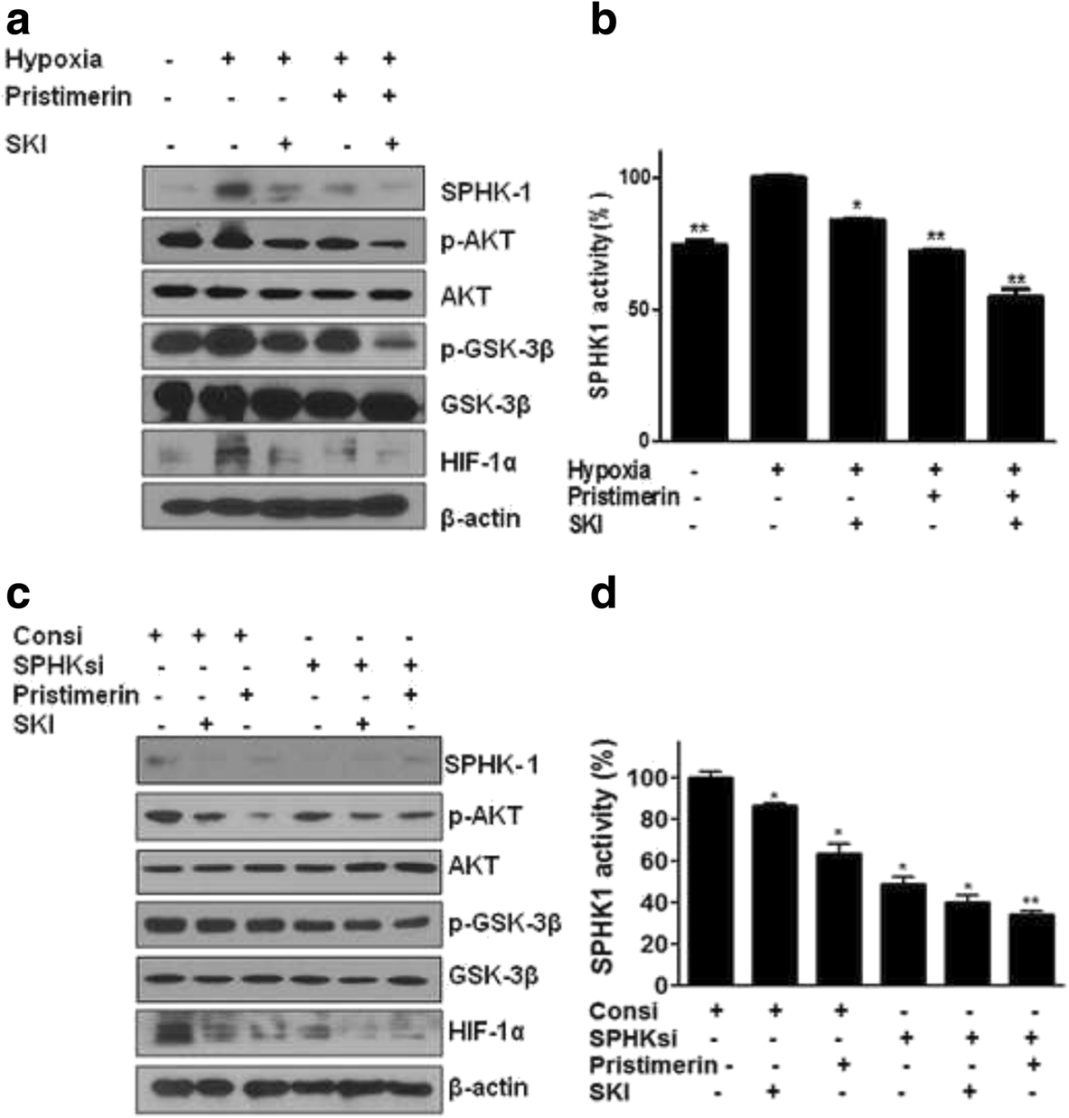
Pristimerin inhibits SPHK-1, and SPHK-1 mediates the activation of HIF-1α under hypoxia. PC-3 cells were treated with pristimerin (1 μM) and or SPHK-1 inhibitor (SKI) (10 μM) for 4 h under hypoxia. **a** Effect of pristimerin on the expression of SPHK-1, HIF-1α, p-AKT and pGSK-3β in hypoxic PC-3 cells. Western blotting was performed to determine the expression of SPHK-1, HIF-1α, p-AKT, AKT, pGSK-3β, GSK-3β, and β-actin in hypoxic PC-3 cells. **b** The activity of SPHK-1 in pristimerin treated PC-3 cells. SPHK-1 activity was measured by using SPHK-1 activity kit. Data are presented as means ± SD. * *p* <0.05 and ** p <0.01 compared with hypoxia control. **c** PC-3 prostate cancer cells were transfected with control vector or SPHK-1 siRNA for 48 h to decrease the expression of SPHK-1. Then PC-3 cells were treated with 1 μM of pristimerin for 4 h. Western blotting was performed to determine the expression of SPHK-1, HIF-1α, p-AKT, AKT, pGSK-3β, GSK-3β, and β-actin in hypoxic PC-3 cells. **d** The activity of SPHK-1 in pristimerin treated PC-3 cells. SPHK-1 activity was measured by using SPHK-1 activity kit. Data are presented as means ± SD. * *p* <0.05 and ** p <0.01 compared with hypoxia control

**Fig. 4:**
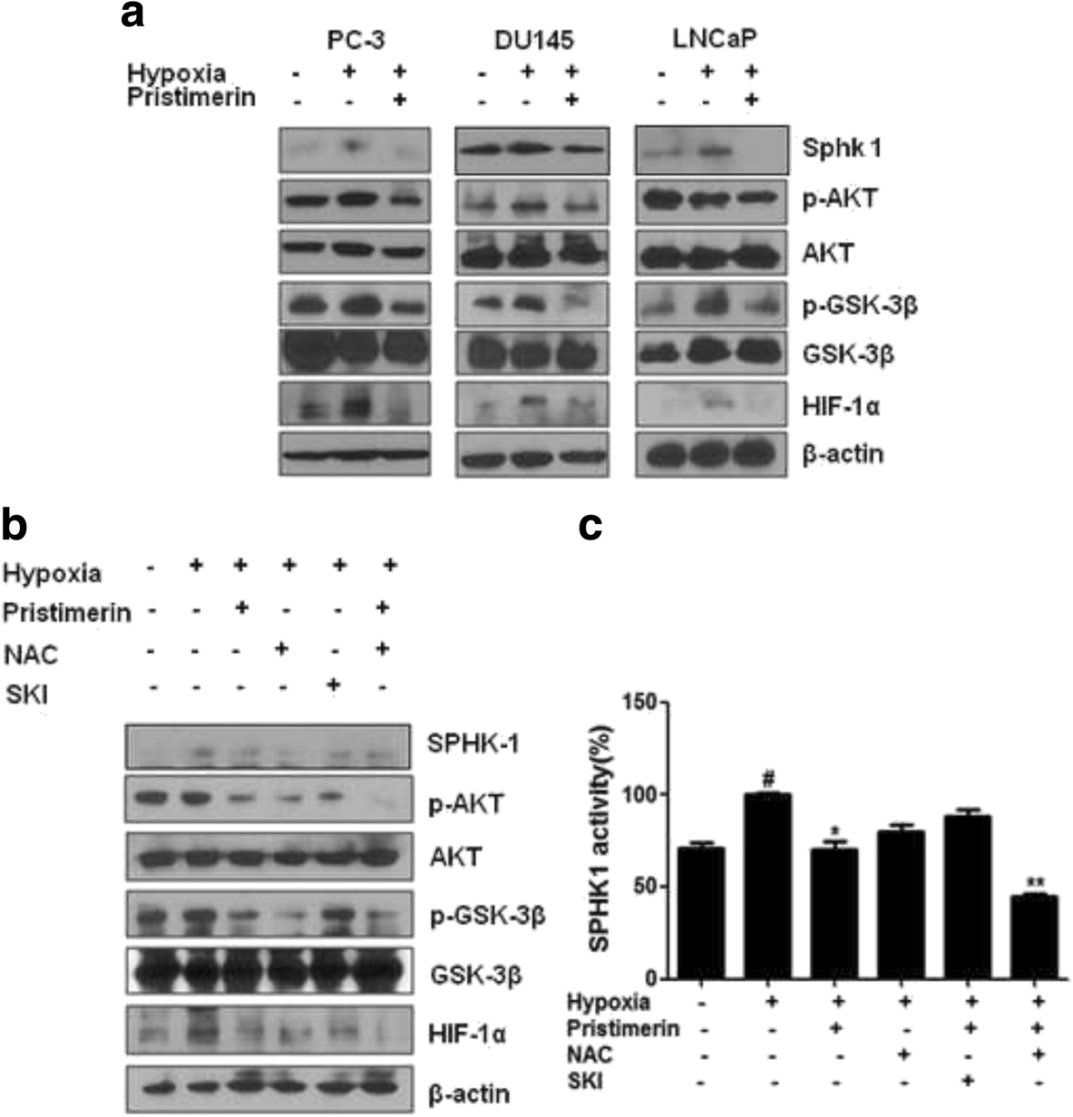
Reactive oxygen species mediate pristimerin inhibited SPHK-1 activity in hypoxic PC-3 cells. Hypoxic PC-3 cells were treated with pristimerin and/or NAC. **a** Western blotting was performed to determine the expression of SPHK-1, HIF-1α, p-AKT, AKT, pGSK-3β, GSK-3β, and β-actin in hypoxic PC-3 cells. **b** SPHK-1 activity in pristimerin and/or NAC-treated PC-3 cells under hypoxia. Data are presented as means ± SD. * *p* <0.05 and ** p <0.01 compared with hypoxia control. *# p* <0.05 compared with normoxia control. **c** Pristimerin suppresses p-AKT and p-GSK-3β via SPHK-1 inhibition in prostate cancer cell lines under hypoxia. Western blotting was performed to determine the expression of SPHK-1, HIF-1α, p-AKT, AKT, pGSK-3β, GSK-3β, and β-actin in hypoxic PC-3 cells

To confirm the involvement of SPHK-1 in the pristimerin-mediated inhibition of HIF-1α during hypoxia, the effects of pristimerin was evaluated by using SPHK-1 siRNA and an SPHK-1 inhibitor, on SPHK-1 activity and the phosphorylation of AKT and GSK-3β. This is because the SPHK-1 dependent stabilization of HIF-1α is known to be mediated by AKT/GSK-3β, downstream of SPHK-1. The phosphorylation of AKT and GSK-3β was induced under hypoxia (Fig. 3a). Pristimerin suppressed the phosphorylation of GSK-3β and AKT in hypoxic PC-3 cells (Fig. 3a). SKI, an SPHK-1 inhibitor, blocked the expression of HIF-1α and the phosphorylation of AKT and GSK-3β (Fig. 3a). The SPHK-1 activity was significantly decreased by pristimerin and SKI (Fig. 3b). Consistently, SPHK-1 siRNA transfection suppressed pristimerin-mediated inhibition of SPHK-1 in PC-3 cells under hypoxia (Fig. 3c and d). As shown in Fig. 4a, we assessed whether pristimerin suppresses hypoxia-induced HIF-1α and SPHK-1 in several prostate cancer cell lines (PC-3, DU145, and LNCaP). Pristimerin inhibited HIF-1α and the phosphorylation of AKT and GSK-3β in all cell lines tested, which is similar to the results from PC-3 cells (Fig. 4a).

### ROS mediates pristimerin inhibited SPHK-1 and HIF-1α in hypoxic PC-3 cells

To examine whether ROS mediate pristimerin-induced inhibition of HIF-1α and SPHK-1, PC-3 cells were treated with pristimerin or/and NAC. The treatment with either pristimerin or NAC reduced hypoxia-mediated HIF-1α, and SPHK-1 expressions and activity (Fig. 4b and c).

### Pristimerin inhibits VEGF production via SPHK-1 inhibition in Hypoxic PC-3 cells

**Fig. 5:**
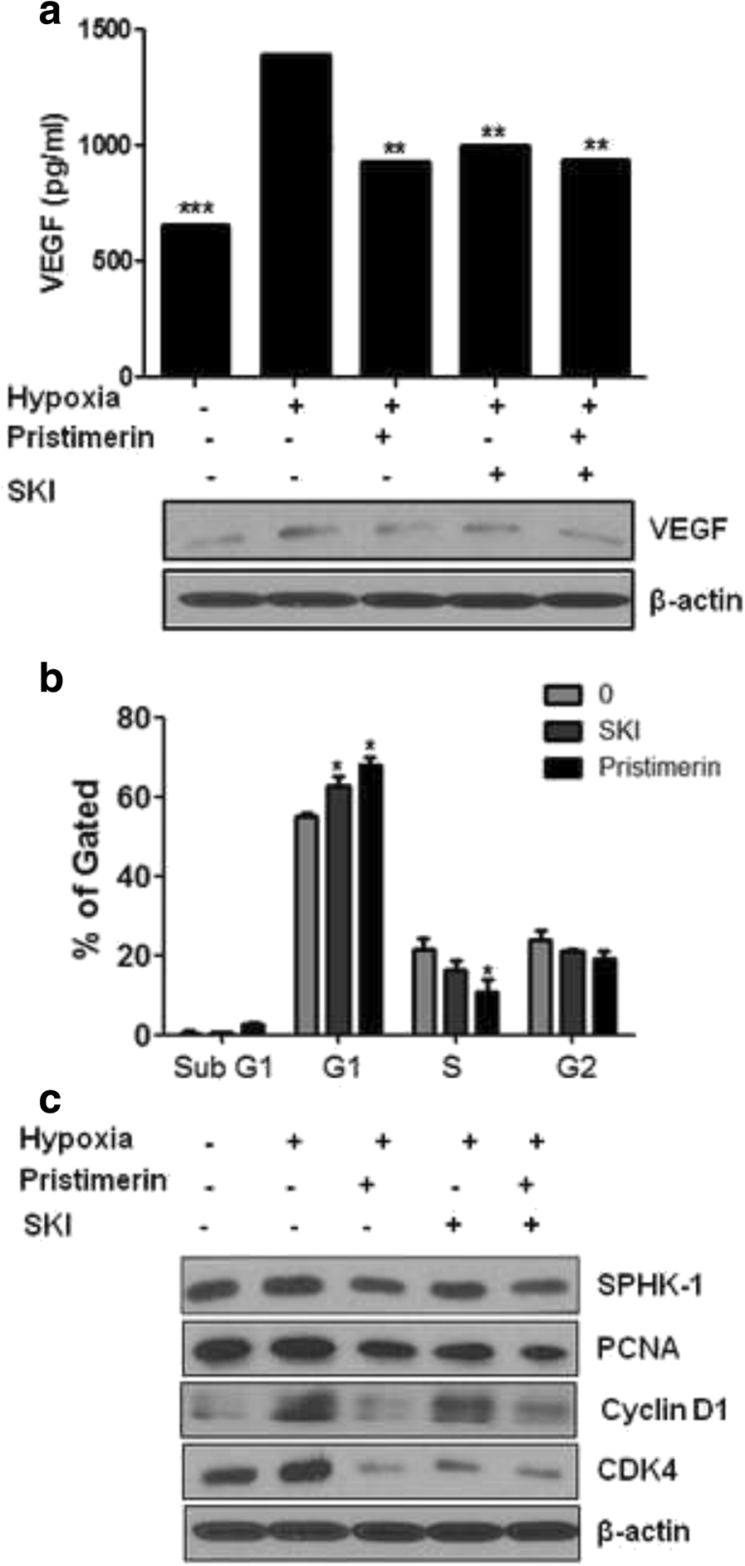
Pristimerin inhibits cell proliferation and VEGF production via SPHK-1 inhibition in PC-3 cells under hypoxia. **a** Cells were treated with pristimerin (1 μM) and/or SPHK-1 inhibitor (SKI) (10 μM) for 24 h under hypoxia. VEGF level was measured by ELISA. Data are presented as means ± SD. ** *p* <0.01 and *** p <0.001 compared with hypoxia control. Western blotting was performed to determine the expression of VEGF and β-actin in hypoxic PC-3 cells. **b** Cells were treated with pristimerin (1 μM) and/or SPHK-1 inhibitor (SKI) (10 μM) for 48 h under hypoxia. Cell cycle distribution was analyzed by flow cytometry. Bar graphs represent the percentage of sub-G1, G1, S, and G2-M phase cells. Data represent mean ± SD of three independent experiments. * *p* <0.05 compared with untreated control. **c** Western blotting was performed to determine the expression of SPHK-1, PCNA, CyclinD1, CDK4, and β-actin in hypoxic PC-3 cells

As shown in Fig. 1c, pristimerin significantly reduced VEGF production. To exam the role of SPHK-1 on the secretion of VEGF, an angiogenic factor, PC-3 cells were treated with pristimerin and SKI under hypoxia for 24 h and VEGF levels were then measured by an ELISA and Western blot. VEGF levels elevated significantly in the hypoxia control group while pristimerin and SKI treatment reduced VEGF secretion (Fig. 5a). In addition, combination treatment with pristimerin and SKI significantly diminished VEGF secretion in PC-3 cells under hypoxia (Fig. 5a).

### SPHK-1 mediates pristimerin-induced G1 arrest in hypoxia-induced PC-3 cells

As shown in Fig. 1b and c, pristimerin significantly decreased cell viability under hypoxia as opposed to normoxia and decreased cell proliferation. Therefore, the effect of SKI and pristimerin on cell proliferation during hypoxia was evaluated by FACS analysis and western blotting.

PC-3 cells were treated with SKI and pristimerin for 48 h under hypoxic conditions. Treatment with pristimerin and SKI significantly increased G1-arrest and decreased the expression of G1 regulatory proteins, such as cylinD1 and CDK4, in hypoxic PC-3 cells (Fig. 5b and c). PCNA is essential for DNA replication. The PCNA level under normoxia was similar to that under hypoxia. SKI and pristimerin treatment reduced the PCNA level. Combination treatment with pristimerin and SKI reduced PCNA under hypoxia (Fig. 5c).

## Discussion

Most solid tumours are more aggressive and resistant to chemotherapy or radiation under hypoxic conditions [37, 41]. Hypoxia is a typical characteristic of locally advanced solid tumours [42]. The transcription factor HIF-1α, which targets 60 genes to enhance the tumour progression, angiogenesis, and metastasis, is regarded as the master regulator under the hypoxic environment [12, 43]. Our previous study showed that the accumulation of HIF-1α is mediated by the AKT/GSK-3β pathway, and related to HIF-1α stabilization through the activation of SPHK-1 [44]. SPHK-1 is a decisive regulator of this sphingolipid rheostat and as such, a potent therapeutic target for cancer treatment [45, 46]. Furthermore, the activity and expression of SPHK-1 are significantly induced under hypoxia and by HIF-1α, and thus is a critical therapeutic target through pVHL-dependent proteasomal degradation for cancer treatment [23, 47–49]. Pristimerin, a triterpenoid quinone methide compound, is involved in a variety of activities, which includes anti-inflammatory and anti-cancer action [27, 29–36]. A recent study reported that pristimerin suppressed HIF-1α and hypoxia-induced metastasis in prostate cancer PC-3 cells [4]. However, the mechanisms of the inhibition of hypoxia-induced HIF-1α by pristimerin are not fully comprehended. In this study, pristimerin significantly decreased cell viability under hypoxia more than it did under normoxia, which connotes the potential of pristimerin treatment-resistant cancer cells, given that HIF-1α promotes cancer resistance. Our study showed that SPHK-1 and HIF-1α accumulations began to increase after 30 min of hypoxia exposure in PC-3 prostate cancer cells compared with the normoxia, which is consistent with previous studies [37, 38]. Moreover, the hypoxia-induced HIF-1α accumulation was suppressed in the presence of pristimerin. In addition, we found that pristimerin suppressed hypoxia-induced SPHK-1. To further confirm the involvement of SPHK-1 in pristimerin-mediated inhibition of HIF-1α under hypoxia, we tested the effects of pristimerin on the phosphorylation of AKT and GSK-3β since AKT/GSK-3β is downstream of SPHK-1 and mediates HIF-1α stabilization [23]. Furthermore, co-treatment of pristimerin and SKI suppressed the phosphorylation of AKT and GSK-3β. Likewise, SPHK-1 siRNA transfection suppressed the phosphorylation of AKT and GSK-3β.

Hypoxia leads to an increase in mitochondrial production of ROS, [50] and ROS production is required for hypoxia-mediated HIF stabilization [51–54]. Several recent studies have shown that SPHK-1 activity and HIF-1α are stimulated by ROS production [44, 55]. In addition, our previous studies showed that *N*-acetylcysteine (NAC), an ROS scavenger, suppresses HIF-1α by blocking SPHK-1 under hypoxia.

To confirm whether pristimerin suppresses hypoxia-induced HIF-1α accumulation via the inhibition of SPHK-1 and ROS generation in prostate cancer cells, we evaluated the effect of NAC on HIF-1α and SPHK-1 abundance in hypoxic PC-3 cells, treated with pristimerin. The co-treatment of pristimerin with NAC affected HIF-1α and SPHK-1 abundance.

PI3K is necessary for cell growth and survival, and PI3K can be activated by growth factors binding to cell surface receptor and hypoxia. PI3K induces the accumulation, activation, and stabilization of HIF-1α proteins during hypoxia in cancer cells [56]. To confirm whether pristimerin inhibits hypoxia-induced HIF-1α accumulation by the inhibition of PI3K, PC-3 cells were treated with pristimerin and SKI under normoxic and hypoxic conditions for 24 h. PI3K levels did not change (Additional file 1: Figure S1).

There is evidence that HIF-1α can regulate VEGF secretion in cancer cells [57, 58]. In the present study, the inhibition of SPHK-1 activity using SKI prevented VEGF production in PC-3 cells. Similarly, studies have demonstrated that SPHK-1 plays a critical role in HIF-1α-mediated VEGF secretion under hypoxia [37, 38]. Pristimerin significantly inhibited cell proliferation for 48 h (Fig. 1c). It is well known that SPHK-1 mediates cancer cell proliferation and progression. Thus, to confirm the involvement of SPHK-1 in pristimerin-mediated inhibition of cell proliferation, hypoxic PC-3 cells were treated with SKI and pristimerin for 48 h. Interestingly, SKI and pristimerin co-treatment induced G1 arrest and decreased G1 regulatory factors in hypoxic PC-3 cells.

## Conclusions

Our study shows that pristimerin inhibits HIF-1α, SPHK-1 expression or activity, and phospho-AKT/GSK-3β and decreases VEGF production in hypoxic PC-3 cells. These results suggest that pristimerin may inhibit HIF-1α accumulation by inactivation of SPHK-1 including the free radical scavenging effect in PC-3 cells under hypoxia.

## Additional file

Additional file 1: Figure S1. Pristimerin does not affect PI3K in PC-3 cells under hypoxia. PC-3 cells were treated with pristimerin (1 μM) and or SPHK-1 inhibitor (SKI) (10 μM) for 4 h under hypoxia. Effect of pristimerin on the expression of PI3K in hypoxic PC-3 cells. Western blotting was performed to determine the expression of PI3K and β-actin in hypoxic PC-3 cells. (TIF 66 kb)

## Abbreviations

FBS: Fetal bovine serum
GSK-3β: Glycogen synthase kinase-3β
HIF-1α: Hypoxia inducible factor 1α
MTT: 3-(4,5-Dimethylthiazol-2-yl)-2,5-diphenyltetrazolium bromide
ROS: Reactive oxygen species
SPHK-1: Sphingsine kinase 1
VEGF: Vascular endothelial growth factor
VHL: Von Hippel-Lindau

## References

[1] Hockel M, Vaupel P (2001). Tumor hypoxia: definitions and current clinical, biologic, and molecular aspects. J Natl Cancer Inst.

[2] Vaupel P, Mayer A (2007). Hypoxia in cancer: significance and impact on clinical outcome. Cancer Metastasis Rev.

[3] Greco O, Marples B, Joiner MC, Scott SD (2003). How to overcome (and exploit) tumor hypoxia for targeted gene therapy. J Cell Physiol.

[4] Zuo J, Guo Y, Peng X, Tang Y, Zhang X, He P, Li S, Wa Q, Li J, Huang S (2015). Inhibitory action of pristimerin on hypoxiamediated metastasis involves stem cell characteristics and EMT in PC-3 prostate cancer cells. Oncol Rep.

[5] Patiar S, Harris AL (2006). Role of hypoxia-inducible factor-1alpha as a cancer therapy target. Endocr Relat Cancer.

[6] Lawrence YR, Dicker AP (2008). Hypoxia in prostate cancer: observation to intervention. Lancet Oncol.

[7] Wang GL, Jiang BH, Rue EA, Semenza GL (1995). Hypoxia-inducible factor 1 is a basic-helix-loop-helix-PAS heterodimer regulated by cellular O2 tension. Proc Natl Acad Sci U S A.

[8] Jaakkola P, Mole DR, Tian YM, Wilson MI, Gielbert J, Gaskell SJ, von Kriegsheim A, Hebestreit HF, Mukherji M, Schofield CJ (2001). Targeting of HIF-alpha to the von Hippel-Lindau ubiquitylation complex by O2-regulated prolyl hydroxylation. Science.

[9] Ivan M, Kondo K, Yang H, Kim W, Valiando J, Ohh M, Salic A, Asara JM, Lane WS, Kaelin WG (2001). HIFalpha targeted for VHL-mediated destruction by proline hydroxylation: implications for O2 sensing. Science.

[10] Epstein AC, Gleadle JM, McNeill LA, Hewitson KS, O’Rourke J, Mole DR, Mukherji M, Metzen E, Wilson MI, Dhanda A (2001). C. elegans EGL-9 and mammalian homologs define a family of dioxygenases that regulate HIF by prolyl hydroxylation. Cell.

[11] Semenza GL (2002). HIF-1 and tumor progression: pathophysiology and therapeutics. Trends Mol Med.

[12] Semenza GL (2003). Targeting HIF-1 for cancer therapy. Nat Rev Cancer.

[13] Cuvillier O, Pirianov G, Kleuser B, Vanek PG, Coso OA, Gutkind S, Spiegel S (1996). Suppression of ceramide-mediated programmed cell death by sphingosine-1-phosphate. Nature.

[14] Spiegel S, Milstien S (2003). Sphingosine-1-phosphate: an enigmatic signalling lipid. Nat Rev Mol Cell Biol.

[15] Pettus BJ, Bielawski J, Porcelli AM, Reames DL, Johnson KR, Morrow J, Chalfant CE, Obeid LM, Hannun YA (2003). The sphingosine kinase 1/sphingosine-1-phosphate pathway mediates COX-2 induction and PGE2 production in response to TNF-alpha. FASEB J.

[16] Billich A, Bornancin F, Mechtcheriakova D, Natt F, Huesken D, Baumruker T (2005). Basal and induced sphingosine kinase 1 activity in A549 carcinoma cells: function in cell survival and IL-1beta and TNF-alpha induced production of inflammatory mediators. Cell Signal.

[17] Olivera A, Edsall L, Poulton S, Kazlauskas A, Spiegel S (1999). Platelet-derived growth factor-induced activation of sphingosine kinase requires phosphorylation of the PDGF receptor tyrosine residue responsible for binding of PLCgamma. FASEB J.

[18] Shu X, Wu W, Mosteller RD, Broek D (2002). Sphingosine kinase mediates vascular endothelial growth factor-induced activation of ras and mitogen-activated protein kinases. Mol Cell Biol.

[19] Rius RA, Edsall LC, Spiegel S (1997). Activation of sphingosine kinase in pheochromocytoma PC12 neuronal cells in response to trophic factors. FEBS Lett.

[20] Meyer zu Heringdorf D, Lass H, Kuchar I, Alemany R, Guo Y, Schmidt M, Jakobs KH (1999). Role of sphingosine kinase in Ca(2+) signalling by epidermal growth factor receptor. FEBS Lett.

[21] Johnson KR, Becker KP, Facchinetti MM, Hannun YA, Obeid LM (2002). PKC-dependent activation of sphingosine kinase 1 and translocation to the plasma membrane. Extracellular release of sphingosine-1-phosphate induced by phorbol 12-myristate 13-acetate (PMA). J Biol Chem.

[22] French KJ, Schrecengost RS, Lee BD, Zhuang Y, Smith SN, Eberly JL, Yun JK, Smith CD (2003). Discovery and evaluation of inhibitors of human sphingosine kinase. Cancer Res.

[23] Ader I, Brizuela L, Bouquerel P, Malavaud B, Cuvillier O (2008). Sphingosine kinase 1: a new modulator of hypoxia inducible factor 1alpha during hypoxia in human cancer cells. Cancer Res.

[24] Costa PM, Ferreira PM, Bolzani Vda S, Furlan M, de Freitas Formenton Macedo Dos Santos VA, Corsino J, de Moraes MO, Costa-Lotufo LV, Montenegro RC, Pessoa C (2008). Antiproliferative activity of pristimerin isolated from Maytenus ilicifolia (Celastraceae) in human HL-60 cells. Toxicol In Vitro.

[25] Brinker AM, Ma J, Lipsky PE, Raskin I (2007). Medicinal chemistry and pharmacology of genus Tripterygium (Celastraceae). Phytochemistry.

[26] Sassa H, Kogure K, Takaishi Y, Terada H (1994). Structural basis of potent antiperoxidation activity of the triterpene celastrol in mitochondria: effect of negative membrane surface charge on lipid peroxidation. Free Radic Biol Med.

[27] Dirsch VM, Kiemer AK, Wagner H, Vollmar AM (1997). The triterpenoid quinonemethide pristimerin inhibits induction of inducible nitric oxide synthase in murine macrophages. Eur J Pharmacol.

[28] Figueiredo JN, Raz B, Sequin U (1998). Novel quinone methides from Salacia kraussii with in vitro antimalarial activity. J Nat Prod.

[29] Yang H, Landis-Piwowar KR, Lu D, Yuan P, Li L, Reddy GP, Yuan X, Dou QP (2008). Pristimerin induces apoptosis by targeting the proteasome in prostate cancer cells. J Cell Biochem.

[30] Yan YY, Bai JP, Xie Y, Yu JZ, Ma CG (2013). The triterpenoid pristimerin induces U87 glioma cell apoptosis through reactive oxygen species-mediated mitochondrial dysfunction. Oncol Lett.

[31] Lee JS, Yoon IS, Lee MS, Cha EY, Thuong PT, Diep TT, Kim JR (2013). Anticancer activity of pristimerin in epidermal growth factor receptor 2-positive SKBR3 human breast cancer cells. Biol Pharm Bull.

[32] Wu CC, Chan ML, Chen WY, Tsai CY, Chang FR, Wu YC (2005). Pristimerin induces caspase-dependent apoptosis in MDA-MB-231 cells via direct effects on mitochondria. Mol Cancer Ther.

[33] Lu Z, Jin Y, Chen C, Li J, Cao Q, Pan J (2010). Pristimerin induces apoptosis in imatinib-resistant chronic myelogenous leukemia cells harboring T315I mutation by blocking NF-kappaB signaling and depleting Bcr-Abl. Mol Cancer.

[34] Wang Y, Zhou Y, Zhou H, Jia G, Liu J, Han B, Cheng Z, Jiang H, Pan S, Sun B (2012). Pristimerin causes G1 arrest, induces apoptosis, and enhances the chemosensitivity to gemcitabine in pancreatic cancer cells. PLoS One.

[35] Mu XM, Shi W, Sun LX, Li H, Wang YR, Jiang ZZ, Zhang LY (2012). Pristimerin inhibits breast cancer cell migration by up- regulating regulator of G protein signaling 4 expression. Asian Pac J Cancer Prev.

[36] Mu X, Shi W, Sun L, Li H, Jiang Z, Zhang L (2012). Pristimerin, a triterpenoid, inhibits tumor angiogenesis by targeting VEGFR2 activation. Molecules.

[37] Cho SY, Lee HJ, Jeong SJ, Lee HJ, Kim HS, Chen CY, Lee EO, Kim SH (2011). Sphingosine kinase 1 pathway is involved in melatonin-induced HIF-1alpha inactivation in hypoxic PC-3 prostate cancer cells. J Pineal Res.

[38] Cho SY, Cho S, Park E, Kim B, Sohn EJ, Oh B, Lee EO, Lee HJ, Kim SH (2014). Coumestrol suppresses hypoxia inducible factor 1alpha by inhibiting ROS mediated sphingosine kinase 1 in hypoxic PC-3 prostate cancer cells. Bioorg Med Chem Lett.

[39] Park KY, Lee HJ, Jeong SJ, Lee HJ, Kim HS, Kim SH, Lim S, Kim HC, Lu J, Kim SH (2010). 1,2,3,4,6-Penta-O-galloly-beta-D-glucose suppresses hypoxia-induced accumulation of hypoxia-inducible factor-1alpha and signaling in LNCaP prostate cancer cells. Biol Pharm Bull.

[40] Cho SM, Lee EO, Kim SH, Lee HJ (2014). Essential oil of Pinus koraiensis inhibits cell proliferation and migration via inhibition of p21-activated kinase 1 pathway in HCT116 colorectal cancer cells. BMC Complement Altern Med.

[41] Melillo G (2007). Targeting hypoxia cell signaling for cancer therapy. Cancer Metastasis Rev.

[42] Vaupel P (2004). The role of hypoxia-induced factors in tumor progression. Oncologist.

[43] Semenza GL (2000). HIF-1: mediator of physiological and pathophysiological responses to hypoxia. J Appl Physiol.

[44] Ader I, Malavaud B, Cuvillier O (2009). When the sphingosine kinase 1/sphingosine 1-phosphate pathway meets hypoxia signaling: new targets for cancer therapy. Cancer Res.

[45] Cuvillier O (2008). Downregulating sphingosine kinase-1 for cancer therapy. Expert Opin Ther Targets.

[46] Cuvillier O (2007). Sphingosine kinase-1--a potential therapeutic target in cancer. Anti-Cancer Drugs.

[47] Yun JK, Kester M (2002). Regulatory role of sphingomyelin metabolites in hypoxia-induced vascular smooth muscle cell proliferation. Arch Biochem Biophys.

[48] Tao R, Zhang J, Vessey DA, Honbo N, Karliner JS (2007). Deletion of the sphingosine kinase-1 gene influences cell fate during hypoxia and glucose deprivation in adult mouse cardiomyocytes. Cardiovasc Res.

[49] Skuli N, Monferran S, Delmas C, Lajoie-Mazenc I, Favre G, Toulas C, Cohen-Jonathan-Moyal E (2006). Activation of RhoB by hypoxia controls hypoxia-inducible factor-1alpha stabilization through glycogen synthase kinase-3 in U87 glioblastoma cells. Cancer Res.

[50] Hamanaka RB, Chandel NS (2010). Mitochondrial reactive oxygen species regulate cellular signaling and dictate biological outcomes. Trends Biochem Sci.

[51] Chandel NS, McClintock DS, Feliciano CE, Wood TM, Melendez JA, Rodriguez AM, Schumacker PT (2000). Reactive oxygen species generated at mitochondrial complex III stabilize hypoxia-inducible factor-1alpha during hypoxia: a mechanism of O2 sensing. J Biol Chem.

[52] Brunelle JK, Bell EL, Quesada NM, Vercauteren K, Tiranti V, Zeviani M, Scarpulla RC, Chandel NS (2005). Oxygen sensing requires mitochondrial ROS but not oxidative phosphorylation. Cell Metab.

[53] Mansfield KD, Guzy RD, Pan Y, Young RM, Cash TP, Schumacker PT, Simon MC (2005). Mitochondrial dysfunction resulting from loss of cytochrome c impairs cellular oxygen sensing and hypoxic HIF-alpha activation. Cell Metab.

[54] Guzy RD, Hoyos B, Robin E, Chen H, Liu L, Mansfield KD, Simon MC, Hammerling U, Schumacker PT (2005). Mitochondrial complex III is required for hypoxia-induced ROS production and cellular oxygen sensing. Cell Metab.

[55] Pchejetski D, Kunduzova O, Dayon A, Calise D, Seguelas MH, Leducq N, Seif I, Parini A, Cuvillier O (2007). Oxidative stress-dependent sphingosine kinase-1 inhibition mediates monoamine oxidase A-associated cardiac cell apoptosis. Circ Res.

[56] Mottet D, Dumont V, Deccache Y, Demazy C, Ninane N, Raes M, Michiels C (2003). Regulation of hypoxia-inducible factor-1alpha protein level during hypoxic conditions by the phosphatidylinositol 3-kinase/Akt/glycogen synthase kinase 3beta pathway in HepG2 cells. J Biol Chem.

[57] Patel NS, Muneer A, Blick C, Arya M, Harris AL (2009). Targeting vascular endothelial growth factor in renal cell carcinoma. Tumour Biol.

[58] Harris AL (2002). Hypoxia--a key regulatory factor in tumour growth. Nat Rev Cancer.

