# Supplementary figures and images for: Anti-cancer effect of pristimerin by inhibition of HIF-1α involves the SPHK-1 pathway in hypoxic prostate cancer cells
Source: BMC Cancer. 2016 Aug 31;16(1):701. doi: 10.1186/s12885-016-2730-2 (PMC5007821; doi:10.1186/s12885-016-2730-2)

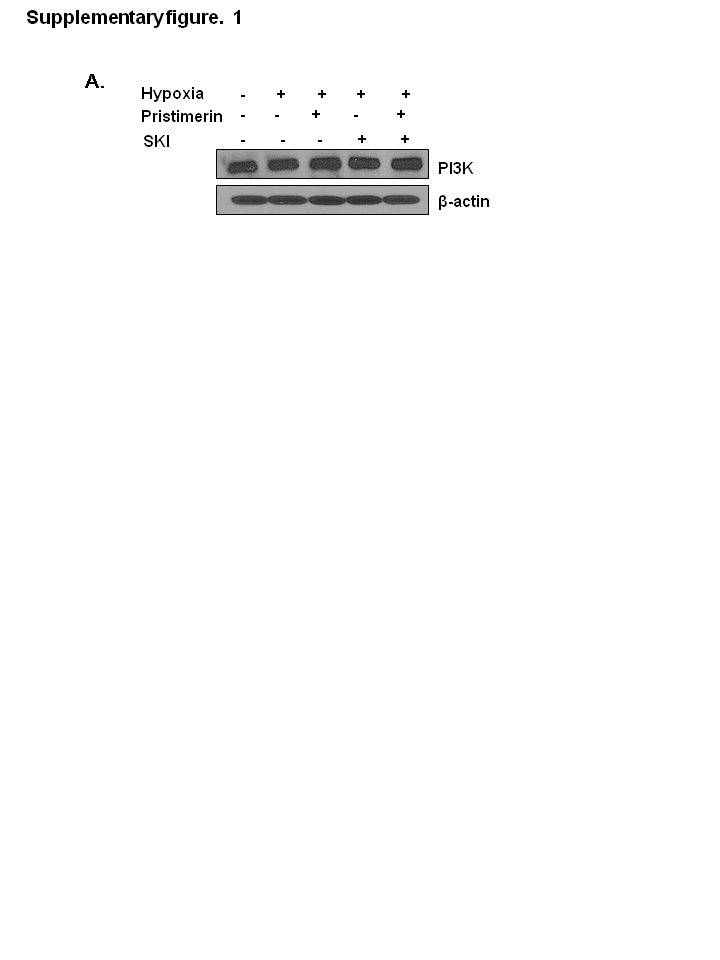

Supplement: Additional file 1: Figure S1. — Pristimerin does not affect PI3K in PC-3 cells under hypoxia. PC-3 cells were treated with pristimerin (1 μM) and or SPHK-1 inhibitor (SKI) (10 μM) for 4 h under hypoxia. Effect of pristimerin on the expression of PI3K in hypoxic PC-3 cells. Western blotting was performed to determine the expression of PI3K and β-actin in hypoxic PC-3 cells. (TIF 66 kb) [file 12885_2016_2730_MOESM1_ESM.tif]
